# Diagnostic performance of cone-beam computed tomography for scaphoid fractures: a systematic review and diagnostic meta-analysis

**DOI:** 10.1038/s41598-021-82351-9

**Published:** 2021-01-28

**Authors:** Ta-Wei Yang, Yen-Yue Lin, Shih-Chang Hsu, Karen Chia-Wen Chu, Chih-Wei Hsiao, Chin-Wang Hsu, Chyi-Huey Bai, Cheng-Kuang Chang, Yuan-Pin Hsu

**Affiliations:** 1 Department of Emergency Medicine, Taoyuan Armed Forces General Hospital, Taoyuan, Taiwan; 2 National Defense Medical Center, Taipei, Taiwan; 3 Emergency Department, Wan Fang Hospital, Taipei Medical University, Taipei, Taiwan; 4 Department of Emergency, School of Medicine, College of Medicine, Taipei Medical University, Taipei, Taiwan; 5 Department of Orthopedic Surgery, Taoyuan Armed Forces General Hospital, Taoyuan, Taiwan; 6 Department of Public Health, School of Medicine, College of Medicine, Taipei Medical University, Taipei, Taiwan; 7 Department of Radiology, Tri-Service General Hospital, National Defense Medical Center, Taipei, Taiwan; 8 Research Center of Big Data and Meta-Analysis, Wan Fang Hospital, Taipei Medical University, Taipei, Taiwan

**Keywords:** Imaging, Skeleton, Trauma

## Abstract

Scaphoid fractures are the most common carpal fractures. Diagnosing scaphoid fractures is challenging. Recently, cone-beam computed tomography (CBCT) has been shown to be a promising strategy for diagnosing scaphoid fractures. The diagnostic performance of CBCT remains inconclusive in the literature. Through a systematic review and meta-analysis, our study aims to determine the diagnostic performance of CBCT for diagnosing scaphoid fractures. Five databases were searched up to March 25, 2020. We included prospective and retrospective studies describing the diagnostic accuracy of CBCT for scaphoid fractures in adult patients. QUADAS-2 tool was used to assess the quality of the included studies. Four studies (n = 350) were included in the meta-analysis. Three of the four studies had high bias risk. The result showed that CBCT had a pooled sensitivity of 0.88 and a pooled specificity of 0.99 for scaphoid fracture diagnosis. The heterogeneities of sensitivity and specificity were substantial. The area under the summary receiver operating characteristic curve was 0.98. No significant publication bias was observed. The result suggested that the diagnostic performance of CBCT for scaphoid fracture was excellent. The certainty of current evidence is low. Further well-designed studies with large sample sizes are warranted to confirm this finding.

## Introduction

Scaphoid fractures are the most common carpal fractures, accounting for 60%–70% of all carpal fractures^1^. The reported incidence is approximately 10.6–29 per 100,000 people per year, with peak occurrence in men between the ages of 20 and 24 years^2^. Among patients with suspected scaphoid fractures, the prevalence of true fractures is estimated to be 5%–20%^3^. Early scaphoid fracture diagnosis is crucial because scaphoid fractures have a high risk of long-term complication, such as nonunion, avascular necrosis, and carpal instability, without timely diagnosis and adequate treatment^4^.

Scaphoid fracture diagnosis has several challenges. Plain radiography is usually used as the first imaging tool; however, its diagnostic accuracy for scaphoid fracture is notoriously poor, with up to 25% unrecognized scaphoid fractures^5^. When radiography shows a negative result or is inconclusive regarding fracture detection, additional cross-sectional imaging can be used to diagnose scaphoid fractures, including ultrasonography (US), computed tomography (CT), magnetic resonance imaging (MRI), and bone scintigraphy (BS). US is more accurate than plain radiography, with a reported pooled sensitivity of 85.6% and specificity of 83.3%^6^. However, US cannot provide clear information on the fracture, such as the extent of dislocation and angulation, which may affect treatment decisions. Moreover, its operator-dependent diagnostic performance is limited^7^. CT is widely used and is more accurate than radiography and US, but it involves considerable radiation exposure^8^. MRI is currently regarded as the reference standard in the literature, but it has a possibility of false positive results and cannot be obtained immediately in most institutions^9^. In a Cochrane meta-analysis, BS showed significantly higher diagnostic accuracy than CT and MRI^10^. However, it is more invasive than other modalities, has safety issues due to high radiation exposure, and has a diagnostic delay of at least 72 h. Therefore, readily available and reliable imaging techniques for scaphoid fracture diagnosis are urgently needed.

Cone-beam computed tomography (CBCT) has been being used in oral and maxillofacial fields for > 20 years, and it provides favorable visualization of details of small bony structures^11^. Compared with traditional or multidetector CT, it requires less space and provides higher spatial resolution under a possibly lower radiation dose exposure. Moreover, it is less invasive and more clinically accessible than MRI and BS. Therefore, using CBCT to diagnose scaphoid fractures is a promising strategy.

Recently, several studies have investigated the use of CBCT for diagnosing scaphoid fractures^12–16^. In these studies, diagnostic performance results have been inconsistent and have not been previously synthesized through a meta-analysis. We thus performed a systematic review and meta-analysis of the diagnostic performance of CBCT for scaphoid fractures.

## Method

We registered our systematic review and meta-analysis protocol on PROSPERO (PROSPERO ID: CRD42020176017). Our study adhered to the Preferred Reporting Items for Systematic Reviews and Meta-Analyses statement^17^. Ethical approval or patient consent was not required as the present study was a review of previously published articles.

We systematically searched PubMed, Embase, Cochrane Library, Web of Science, and Scopus from inception up to March 25, 2020. Search keywords included fracture, scaphoid, wrist, and cone-beam computed tomography. Details of the search are provided in Supplementary Table 1. We included prospective and retrospective studies describing the diagnostic accuracy of CBCT for wrist bone fractures in adult patients (age ≥ 15 years). Reviews, case series, case reports, conference proceedings, and animal studies were excluded. Moreover, studies focusing on soft tissue injury were excluded. No language restriction was applied. Two reviewers (TWY and YYL) screened all titles and abstracts to identify potentially eligible studies independently. The full text of potentially suitable articles was retrieved, and these articles were checked for inclusion by the two reviewers (TWY and YYL) independently. In case of disagreement, a third reviewer (YPH) made the final decision. We conducted the study selection procedure in EndNote, version 17 (Thomson Research Soft, Stamford, CT, USA). Finally, we examined the reference lists of all included studies to identify additional relevant studies.

Two investigators (KCWC and SCH) independently extracted data from the included studies. The following characteristics from each selected study were extracted: first author, publication year, study design, country, inclusion and exclusion criteria, sample size, age, sex, index test, reference standard, and the number of true positive, false positive, false negative, and true negative cases.

Two researchers (TWY and YYL) independently used Quality Assessment of Studies of Diagnostic Accuracy-2 to assess the quality of the included studies^18^. The tool has four domains, namely patient selection, index test, reference standard, and flow and timing. We assessed all domains for risk of bias and the first three domains for applicability concerns. We rated each domain as low risk, unclear risk, and high risk. We summarized the result using Review Manager Version 5.3 (The Nordic Cochrane Centre, The Cochrane Collaboration, Copenhagen, Denmark). Disagreements were resolved through discussion.

We used a bivariate random-effects model to calculate diagnostic test accuracy variables, including sensitivity, specificity, positive likelihood ratio (PLR), negative likelihood ratio (NLR), and diagnostic odds ratio. The area under the summary receiver operating characteristic (SROC) curve was calculated. All data were calculated with 95% confidence intervals (CIs). We assessed the heterogeneity using the chi-square test and I^2^ statistics. P < 0.1 or I^2^ > 50% suggested substantial heterogeneity. The publication bias in meta-analyses of diagnostic test accuracy was assessed using Deek’s funnel plot asymmetry test^19^. We completed the meta-analysis with the MIDAS module for StataMP, version 14 (StataCorp LP, College Station, Texas).

We used Bayes theorem to estimate the posttest probability of scaphoid fracture. The posttest probability was calculated by multiplying pretest odds with the likelihood ratio. The pretest odds are calculated by dividing the pretest probability by (1-pretest probability). On the basis of pooled PLR and NLR, we used Fagan plot analysis to estimate posttest probabilities with the pretest probabilities of 25%, 50%, and 75%, respectively.

## Results

### Study selection

**Figure 1:**
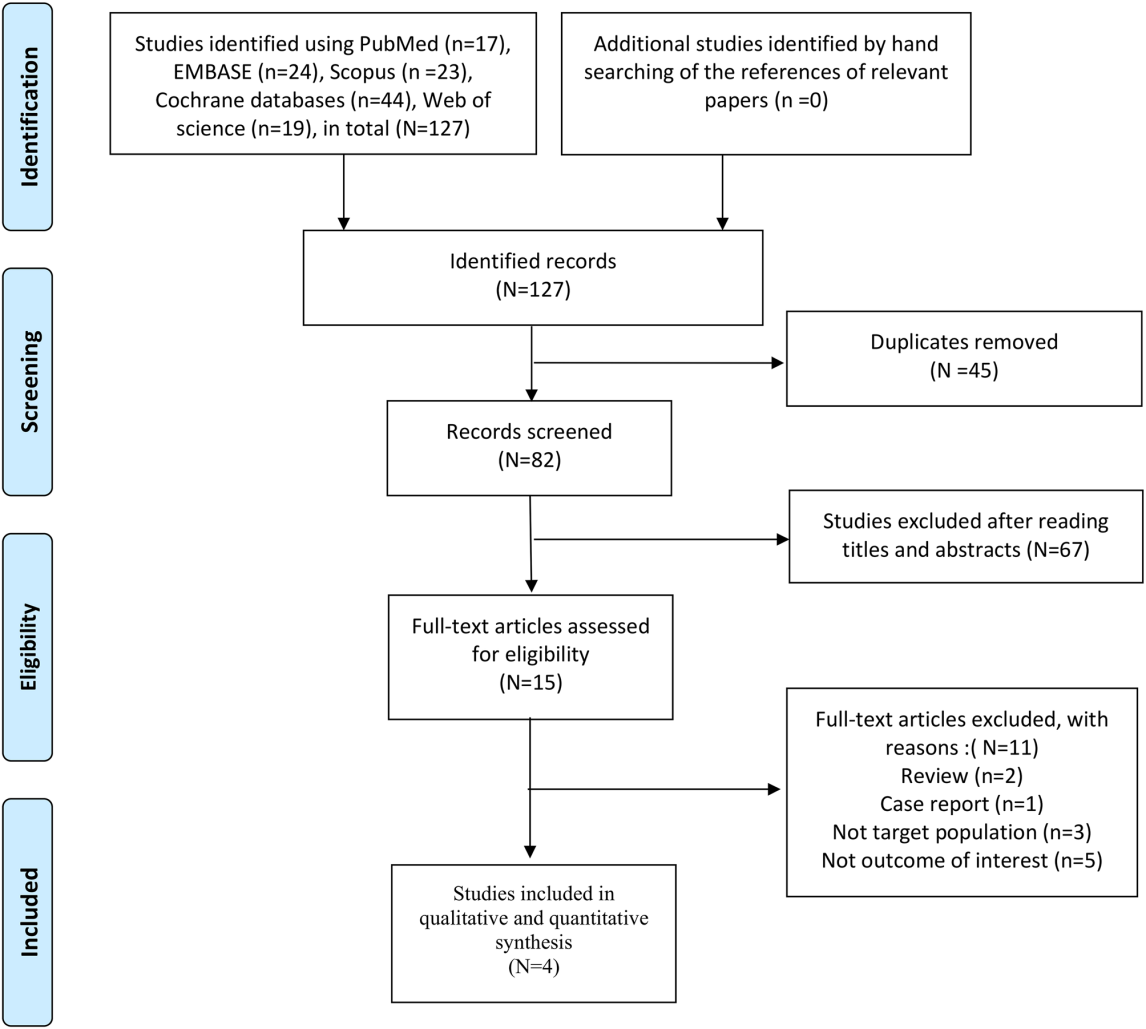
Flowchart of study selection for the current meta-analysis.

The flow diagram of study selection is displayed in Fig. 1. In total, 127 citations were identified after searching relevant databases. After removing duplicate records and excluding irrelevant studies through screening of titles and abstracts, 15 studies were selected in the full-text review stage. Of these, 11 were excluded because they comprised of reviews (n = 2), a case report (n = 1), did not involve the target population (n = 3) and did not examine the outcome of interest (n = 5). Finally, four^12–15^ studies met the inclusion criteria.

### Study characteristics

The characteristics of the studies included in the meta-analysis are summarized in Table 1. All of the four^12–15^ studies were performed in Europe. Three^12,13,15^ were prospective studies, and one^14^ was a retrospective study. The participants mainly included patients aged ≥ 15 years. The mean age of the included participants was between 33 and 41 years. Three^12–14^ studies included patients with clinically suspected scaphoid fractures, and one^15^ study included patients with suspected radiocarpal fractures. The sample sizes ranged from 49 to 117. All studies^12–15^ included more men than women and used CBCT as the index test. The details of the CBCT technique are summarized in Table 2. All studies^12–15^ included MRI as a reference standard; three^12,14,15^ of the studies used multiple reference standards through combination with other imaging modalities.

**Table 1:** Characteristics of included studies.

Study	Country	Design	Inclusion criteria	Sample size	Age, year (mean)	Sex (F/M)	Index test	Reference standard
Gibney^15^	Ireland	Prospective	Suspected radiocarpal fracture but had normal radiographs; age ≥ 16 years; CBCT was performed within 14 days	117	41	57/60	CBCT	Consensus of two radiologists regarding radiography, CBCT, and MRI results
Neubauer^14^	Germany	Retrospective	Suspected scaphoid fracture and had CBCT; age > 18 years	102	33	20/82	CBCT	Consensus of two radiologists regarding radiography, CBCT, MRI results
Borel^13^	France	Prospective	Suspected scaphoid fracture but had normal radiographs; age > 18 years	49	36	18/31	CBCT	MRI
Edlund^12^	Sweden	Prospective	Clinical suspicion of scaphoid fracture; age ≥ 15 years	95	40	38/57	CBCT	CBCT, MRI

**Table 2:** Details of the CBCT protocol.

Study	CBCT scanner (company)	Voltage/current	Slice thickness/increment	Field of view	Matrix	Acquisition time	Processing time
Gibney^15^	Planmed Verity (Planmed)	90 kV/6 mA	0.2 mm/NA	150 × 150 mm	NA	30 s	1 min
Neubauer^14^	Planmed Verity (Planmed)	90 kV/36 mA	0.2 mm/NA	NA	801 × 801	NA	NA
Borel^13^	ProMax 3D mid (Planmeca)	90 kV/120 mA	0.5 mm/NA	90 × 90 mm	NA	15 s	NA
Edlund^12^	Planmed Verity (Planmed)	80–96 kV/6–12 mA	0.2–1 mm/0.4–0.5 mm	130 × 160 mm	NA	36 s	1 min

*NA* not available.

### Bias risk and applicability of the included studies

**Figure 2:**
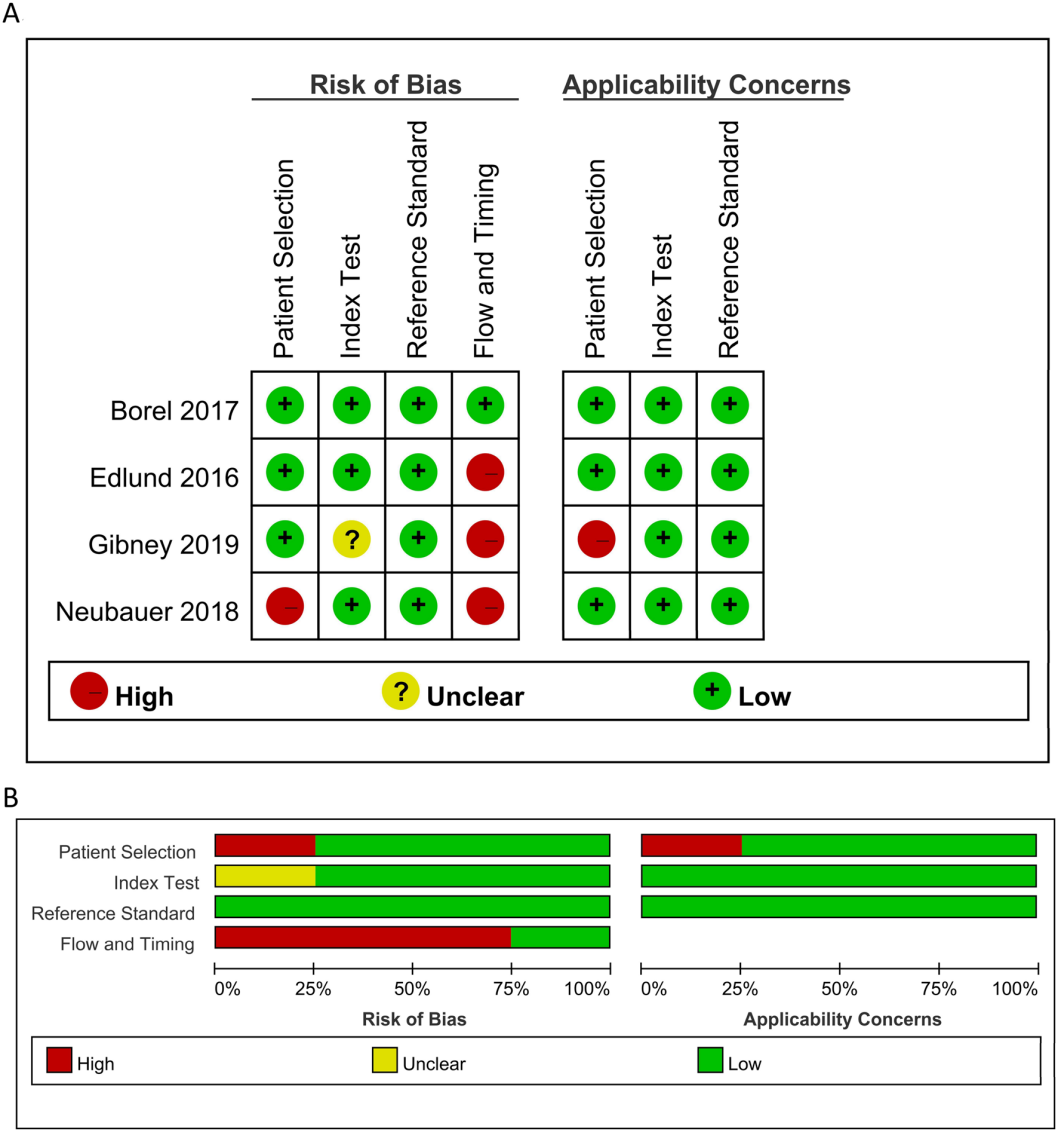
Methodological quality of included studies.

Figure 2 presents the methodological quality assessment of bias risk of eligible studies. One^13^ study was rated as low bias risk and low concern of patient applicability. Three studies^12,14,15^ were rated as high bias risk because, in one^14^ of the three studies, the authors did not consecutively recruit patients suspected of having scaphoid fractures, and not all patients in these three^12,14,15^ studies received the same reference standard. Concerning applicability, one^15^ study used a retrospective design with high concerns of patient applicability.

### Meta-analysis of CBCT for diagnosing scaphoid fractures

**Figure 3:**
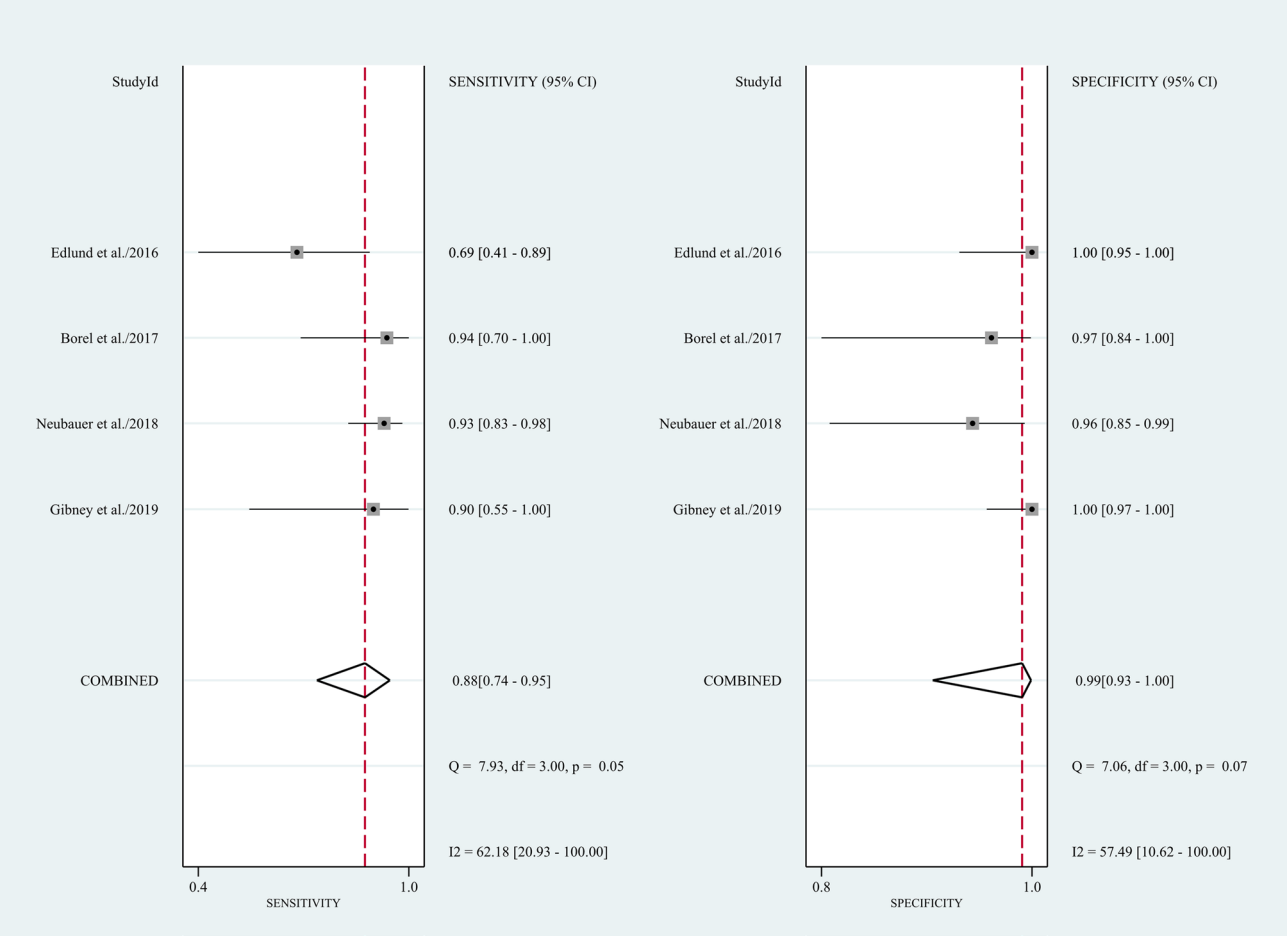
Forest plots of sensitivity and specificity for CBCT in scaphoid fracture diagnosis. Point estimates of sensitivity and specificity from each study are shown as solid circles. Error bars represented as 95% CIs.

**Figure 4:**
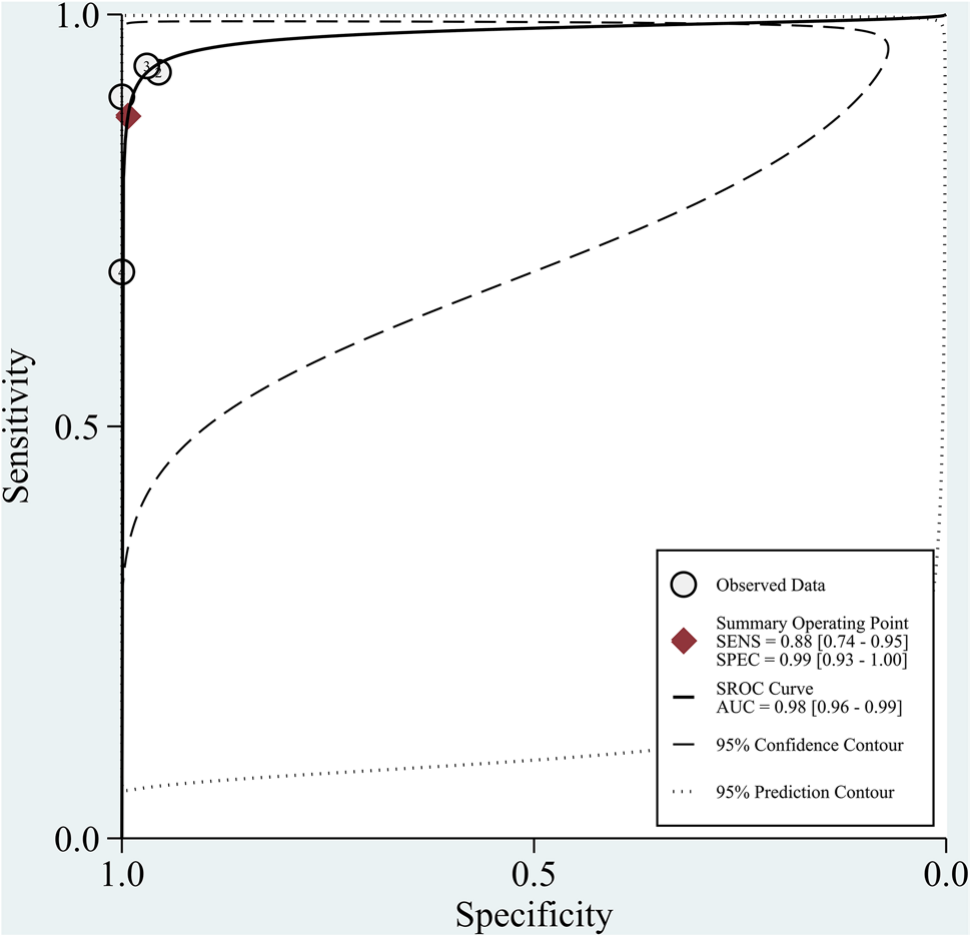
SROC curve for CBCT in scaphoid fracture detection.

**Figure 5:**
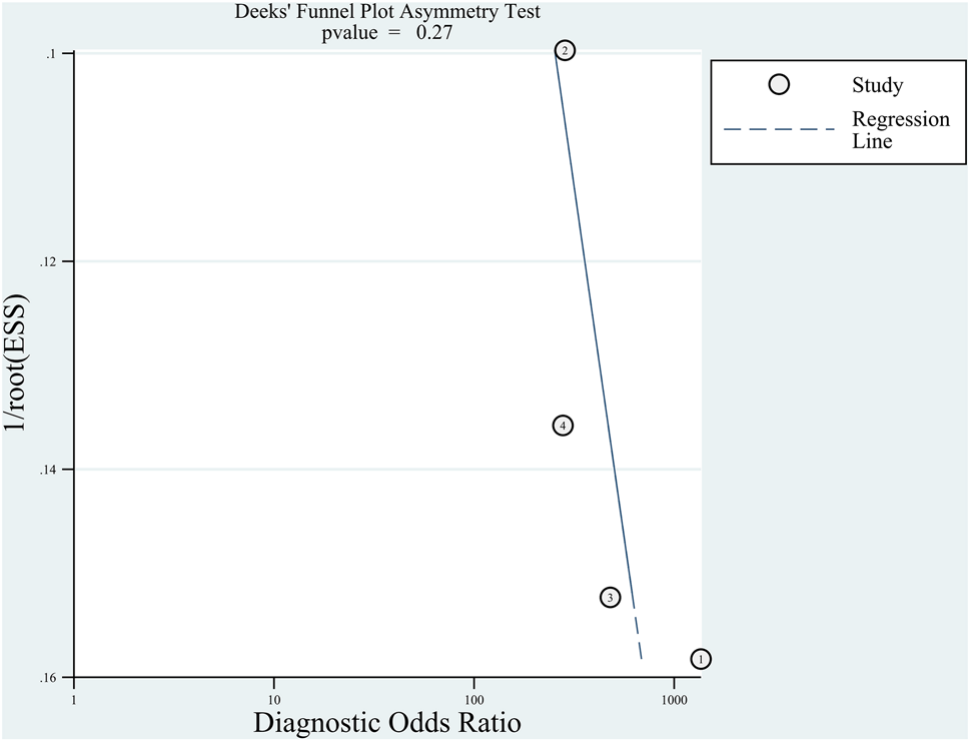
Deek’s funnel plot asymmetry test for publication bias for CBCT.

Four^12–15^ studies (n = 350) were included in the meta-analysis. The result indicated that CBCT had a pooled sensitivity of 0.88 (95% CI  0.74–0.95, I^2^ = 62.18; Fig. 3) and a pooled specificity of 0.99 (95% CI 0.93–1.00, I^2^ = 57.49; Fig. 3) for scaphoid fracture diagnosis. Notably, heterogeneities among studies were substantial. This resulted in a PLR of 119.0 (95% CI 11.7–1210.2; Supplementary Table 2), NLR of 0.12 (95% CI 0.06–0.27; Supplementary Table 2), and Diagnostic odds ratio (DOR) of 957 (95% CI 110–8346; Supplementary Table 2). The area under the SROC curve indicated a high accuracy of CBCT in scaphoid fracture diagnosis (0.98, 95% CI 0.96–0.99; Fig. 4). Funnel plot analysis indicated no significant publication bias (*p* = 0.27; Fig. 5).

## Discussion

To the best of our knowledge, this is the first systematic review and meta-analysis to investigate the diagnostic performance of CBCT for scaphoid fractures. The result of our meta-analysis demonstrated that CBCT has both high sensitivity and high specificity for scaphoid fracture diagnosis.

Scaphoid fracture diagnosis remains challenging for clinicians. Currently, for scaphoid fracture diagnosis, clinical history, physical examination, plain radiography, and further imaging are included. Clinical history^10^, physical examinations^10^, and plain radiography^20^ have insufficient sensitivity and specificity for scaphoid fracture diagnosis. Further cross-sectional imaging is advocated for susceptible patients. A guideline in the United Kingdom suggests BS, CT, and MRI for suspected fractures^21^. Furthermore, another guideline in the United States suggests MRI^22^. A recent meta-analysis showed that MRI has the highest sensitivity and specificity for detecting scaphoid fractures^8^. However, CT is widely used in current practice because it is easily available and costs less than MRI^8^. Mallee et al. showed that CT has a sensitivity of 72% and specificity of 99% for diagnosing scaphoid fractures^10^. In our study, CBCT had a specificity of 99% and sensitivity of 88% for diagnosing scaphoid fractures, which implies that CBCT has higher diagnostic performance than CT.

Recent studies on CBCT have shown that CBCT may replace conventional CT for scaphoid fracture diagnosis^23,24^. Practically, CBCT requires a relatively small space, which makes it easier for medical institutions to retrofit the new space. One of the main differences between CBCT and CT is that CBCT has a higher spatial resolution^25–27^, which makes it a more effective tool for bone visualization^24^. Moreover, CBCT is associated with a lower radiation dose than CT without optimizing the examination protocols^28–30^. Tschauner et al. found that adapted extremity CBCT imaging protocols can fall below optimized pediatric ankle and wrist CT doses at comparable image qualities^31^. Additionally, CBCT showed excellent agreement with CT to confirm fractures in patients with distal limb trauma^32^. Moreover, two studies have demonstrated that CBCT has high interrater agreement with CT for scaphoid fracture diagnosis^13,15^. Furthermore, Honigmann et al. indicated that CBCT is more cost-effective than CT and MRI^33^.

Despite these aforementioned prospects, some disadvantages may limit CBCT application. The acquisition time required by CBCT makes it prone to motion artifact^34^. However, Spin-Neto et al. observed that the automated motion artifact-correction system significantly enhanced CBCT image quality and interpretability^35^. Regarding soft tissue visualization, CBCT performs worse than CT^24^. Moreover, metallic implants produce beam hardening artifacts in CBCT, and sufficient evidence is lacking for CBCT use in the postoperative evaluation of patients with implants^36^. Moreover, CT is diagnostically accurate for scaphoid fracture despite being slightly inferior to CBCT^10,13^. Furthermore, CT shows high diagnostic accuracy for trauma, brain, chest, and abdominal emergency and has wide clinical application^37,38^. Consequently, despite giving a detailed visualization of small bony structures, CBCT only provides a small field of view, which may limit its widespread use. Therefore, in clinical practice, at medical institutions equipped with CBCT, clinicians may consider using CBCT instead of CT for the diagnosis of patients suspected with scaphoid fracture.

Importantly, the pretest probability of the disease may influence the application of CBCT for scaphoid fracture. When we applied a pretest probability of 25%, the posttest probabilities of positive and negative CBCTs were 98% and 4%, respectively; when we applied a pretest probability of 50%, the posttest probabilities of positive and negative CBCTs were 99% and 11%, respectively. When we applied a pretest probability of 75%, the posttest probabilities of positive and negative CBCTs were 99.7% and 27%, respectively (Supplementary Fig. 1). Clinicians could reliably confirm the disease through CBCT with evidence of scaphoid fracture. However, for a negative examination, clinicians had insufficient information to exclude the diagnosis when the scaphoid fracture prevalence was > 50%. In the included studies, the overall prevalence ranged from 8.6% to 52.0%. Two^13,14^ of the included studies in our meta-analysis had a prevalence rate of > 50%, which is higher than that reported 5%–20% in the literature^3^. Thus, different studies included patients with different demographic and clinical factors for the prediction of a true fracture. Duckworth et al. indicated that the predictors of a true scaphoid fracture included male gender, sports injury, anatomical snuffbox pain on the ulnar deviation of the wrist, and pain on thumb-index finger pinch at presentation; predictors at the 2-week review were scaphoid tubercle tenderness^39^. These predictors raised the prevalence of true fractures among suspected fractures. Subsequently, they improved the diagnostic accuracy and lowered the impact on clinical practice. Taken together, the results indicate that in these high-risk patients with a negative CBCT result, a scaphoid fracture cannot be excluded and needs immobilization in a splint.

The timing of testing may have an essential role in the diagnostic accuracy of imaging for scaphoid fracture. Among the included studies in our meta-analysis^12–15^, the timing for applying CBCT varied after the inciting event. CBCT was performed on the first day in Edlund’s study^12^, within 7 days in Borel’s study^13^, within 4 days in Neubauer’s study^14^, and within 7–14 days in Gibney’s study^15^. Nonetheless, Kumar et al. found that the sensitivity of MRI within 24 h after trauma was comparable to day 10 after initial presentation^40^. Moreover, the early use of MRI resulted in low pain and high satisfaction scores as well as less time for immobilization, early treatment, and less time off work and did not increase health costs^41^. Another study indicated that early CT or MRI significantly reduced diagnosis time^42^. Given these potential social benefits, we suggest early CBCT, from 1 to 14 days of injury, to be the management technique of choice. Further studies are needed to clarify the optimal timing for CBCT.

Our study has several limitations. First, the results were based on a limited number of included observational studies with relatively small sample sizes. The precision for summary estimates may be insufficient. Second, three of four included studies were rated as high risk of bias due to the use of multiple reference standards. Third, we found substantial heterogeneities of pooled sensitivity and specificity, which may be attributed to different clinical manifestations of the included participant, different study design, various protocols of CBCT, and the use of different reference standards among studies. Fourth, although we did not detect publication bias, the finding was not powerful because < 10 studies were included. The aforementioned limitations may downgrade the certainty of evidence. Moreover, all studies were conducted in Europe. Therefore, further investigation is required before generalizing these findings to other continents.

In conclusion, the literature suggests that CBCT is both sensitive and specific for scaphoid fracture diagnosis. CBCT exhibits higher diagnostic performance than CT and should be considered as a replacement when the institution is equipped with CBCT. For high-risk patients, a negative result of CBCT should be interpreted with caution. Further studies are needed before routine implementation because of the current low certainty of evidence.

## Supplementary Information

Supplementary Information.

## Data Availability

All data generated or analyzed during this systematic review are included in this published article (and its Supplementary File).
