# Supplementary material for: Diagnostic performance of cone-beam computed tomography for scaphoid fractures: a systematic review and diagnostic meta-analysis
Source: Sci Rep. 2021 Jan 28;11:2587. doi: 10.1038/s41598-021-82351-9 (PMC7843979; doi:10.1038/s41598-021-82351-9)
Supplement: Supplementary file 1 — Supplementary Information. [file 41598_2021_82351_MOESM1_ESM.pdf]

Diagnostic performance of cone-beam computed tomography for scaphoid fractures—A systematic review and diagnostic meta-analysis

Authors

Ta-Wei Yang, Yen-Yue Lin, Shih-Chang Hsu, Karen Chia-Wen Chu, Chih-Wei Hsiao, Chin-Wang Hsu, Chyi-Huey Bai, Cheng-Kuang Chang, Yuan-Pin Hsu

Supplementary Online Content

Table 1 Search Strategy
Table 2 Summary performance estimates of diagnostic parameters
Figure 1 Analysis of Fagan plot to evaluate the clinical utility of cone-beam computed tomography for detecting scaphoid fracture

Table 1. Search strategy

Recent queries in PubMed on March 25, 2020		
**Search**	**Query**	**Items found**
1	Search cone beam	15492
2	Search cone-beam	14773
3	Search scaphoid	5028
4	Search "scaphoid bone"[MeSH Terms]	2185
5	Search wrist	43688
6	Search "wrist joint"[MeSH Terms]	10087
7	Search "wrist"[MeSH Terms]	8870
8	Search radiocarpal	1455
9	Search fracture*	301881
10	Search fracture	279410
11	Search "fractures, bone"[MeSH Terms]	181784
12	Search CBCT	8550
13	Search computed tomography	578061
14	Search "tomography, x-ray computed"[MeSH Terms]	424374
15	Search ((cone beam) OR cone-beam) OR CBCT	16002
16	Search (computed tomography) OR "tomography, x-ray computed"[MeSH Terms]	578061
17	Search (((computed tomography) OR "tomography, x-ray computed"[MeSH Terms])) AND (((cone beam) OR cone-beam) OR CBCT)	14059
18	Search (((((scaphoid) OR "scaphoid bone"[MeSH Terms]) OR wrist) OR "wrist joint"[MeSH Terms]) OR "wrist"[MeSH Terms]) OR radiocarpal	45958
19	Search ((fracture*) OR fracture) OR "fractures, bone"[MeSH Terms]	304052
20	Search (((((((scaphoid) OR "scaphoid bone"[MeSH Terms]) OR wrist) OR "wrist joint"[MeSH Terms]) OR "wrist"[MeSH Terms]) OR radiocarpal)) AND (((fracture*) OR fracture) OR "fractures, bone"[MeSH Terms])	10553
21	Search (((((((((scaphoid) OR "scaphoid bone"[MeSH Terms]) OR wrist) OR "wrist joint"[MeSH Terms]) OR "wrist"[MeSH Terms]) OR radiocarpal)) AND (((fracture*) OR fracture) OR "fractures, bone"[MeSH Terms]))) AND ((((computed	17

	tomography) OR "tomography, x-ray computed"[MeSH Terms])) AND (((cone beam) OR cone-beam) OR CBCT))	
**Recent queries in Web of Science on March 25, 2020**		
**Search**	**Query**	**Items found**
1	TOPIC: (cone beam)	16,517
2	TOPIC: (cone-beam)	13,476
3	TOPIC: (CBCT)	7,871
4	TOPIC: (computed tomography)	261,607
5	TOPIC: (wrist)	31,555
6	TOPIC: (scaphoid)	2,941
7	TOPIC: (radiocarpal)	949
8	TOPIC: (fracture*)	405,128
9	#3 OR #2 OR #1	18,122
10	#9 AND #4	9,986
11	#7 OR #6 OR #5	33,207
12	#11 AND #8	6,050
13	#12 AND #10	19
**Recent queries in Scopus on March 25, 2020**		
**Search**	**Query**	**Items found**
1	TITLE-ABS-KEY (cone AND beam)	27344
2	TITLE-ABS-KEY (cone-beam)	22163
3	TITLE-ABS-KEY (cbct)	11381
4	TITLE-ABS-KEY (computed AND tomography)	642319
5	(TITLE-ABS-KEY (cone AND beam) OR TITLE-ABS-KEY (cone-beam) OR TITLE-ABS-KEY (cbct))	28439
6	TITLE-ABS-KEY (computed AND tomography) AND (TITLE-ABS-KEY (cone AND beam) OR TITLE-ABS-KEY (cone-beam) OR TITLE-ABS-KEY (cbct))	18983
7	TITLE-ABS-KEY (scaphoid)	6707
8	TITLE-ABS-KEY (wrist)	65263
9	TITLE-ABS-KEY (radiocarpal)	1633
10	TITLE-ABS-KEY (fracture*)	789632
11	TITLE-ABS-KEY (scaphoid) OR TITLE-ABS-KEY (wrist) OR TITLE-ABS-KEY (radiocarpal)	68158

12	(TITLE-ABS-KEY (scaphoid) OR TITLE-ABS-KEY (wrist) OR TITLE-ABS-KEY (radiocarpal)) AND TITLE-ABS-KEY (fracture*)	14375
13	((TITLE-ABS-KEY (scaphoid) OR TITLE-ABS-KEY (wrist) OR TITLE-ABS-KEY (radiocarpal)) AND TITLE-ABS-KEY(fracture*)) AND (TITLE-ABS-KEY (computed AND tomography) AND (TITLE-ABS-KEY (cone AND beam) OR TITLE-ABS-KEY (cone-beam) OR TITLE-ABS-KEY (cbct)))	23
**Recent queries in Embase on March 25, 2020**		
**Search**	**Query**	**Items found**
1	cone AND beam	21654
2	'cone beam'	20636
3	cbct	12570
4	computed AND tomography	504777
5	#1 OR #2 OR #3	23924
6	#4 AND #5	20006
7	scaphoid	6463
8	wrist	62364
9	radiocarpal	1922
10	fracture*	404642
11	#7 OR #8 OR #9	65219
12	#10 AND #11	14107
13	#6 AND #12	24
**Recent queries in Cochrane Library on March 25, 2020**		
**Search**	**Query**	**Items found**
1	cone beam	873
2	cone-beam	853
3	cbct	656
4	computed tomography	15255
5	#1 or #2	873
6	#5 and #4	737
7	#6 or #3	1031
8	scaphoid	197
9	wrist	5460

10	radiocarpal	49
11	fracture*	22012
12	MeSH descriptor: [Fractures, Bone] explode all trees	5777
13	#8 or #9 or # 10	936641
14	#11 or #12	22036
15	#13 and #14	14499
16	#7 and #15	44

Table 2. Summary performance estimates of diagnostic parameters

Parameter	Point estimate	95% CI
Sensitivity	0.88	0.74-0.95
Specificity	0.99	0.93-1.00
PLR	119.0	11.7-1210.2
NLR	0.12	0.06-0.27
DOR	957	110-8346

Footnote: CI, confidence interval; DOR, diagnostic odds ratio; NLR, negative likelihood ratio; PLR, positive likelihood ratio

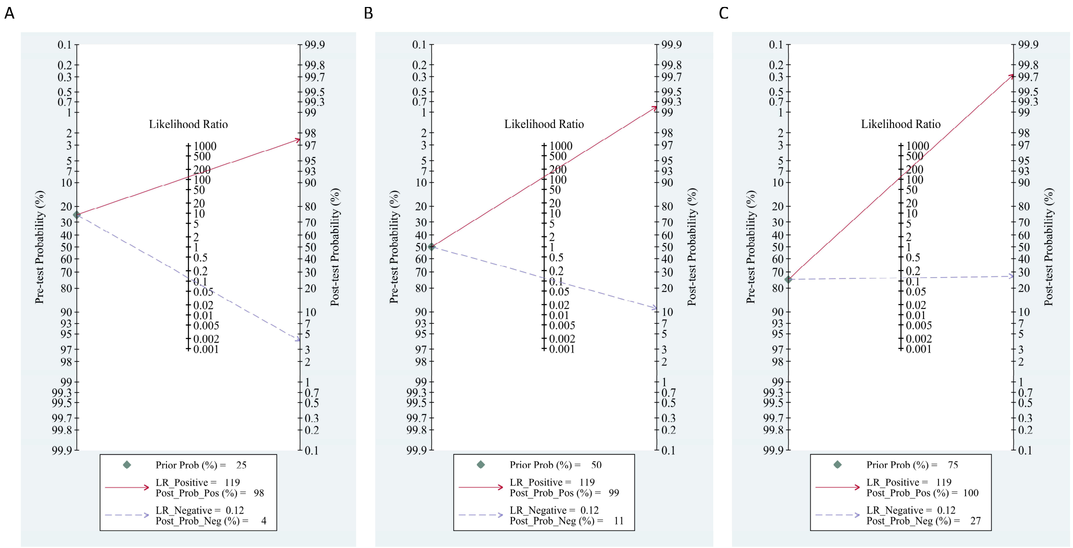

Figure 1. Analysis of Fagan plot to evaluate the clinical utility of cone-beam computed tomography for detecting scaphoid fracture. (A) Pretest probability of 25% for scaphoid fractures. (B) Pretest probability of 50% for scaphoid fractures. (C) Pretest probability of 75% for scaphoid fractures. Fagan plot consists of a vertical axis on the left showing pretest probability, an axis in the middle representing the likelihood ratio, and a vertical axis on the right showing posttest probability. NLR, negative likelihood ratio; PLR, positive likelihood ratio.
